# Activity of the Di-Substituted Urea-Derived Compound I-17 in *Leishmania* In Vitro Infections

**DOI:** 10.3390/pathogens13020104

**Published:** 2024-01-24

**Authors:** José Vitorino dos Santos, Jorge Mansur Medina, Karina Luiza Dias Teixeira, Daniel Marcos Julio Agostinho, Michael Chorev, Aurora Diotallevi, Luca Galluzzi, Bertal Huseyin Aktas, Ulisses Gazos Lopes

**Affiliations:** 1 Laboratory of Molecular Parasitology, Instituto de Biofísica Carlos Chagas Filho, Universidade Federal do Rio de Janeiro (UFRJ), Rio de Janeiro 21941-902, Brazil; josevitorino@biof.ufrj.br (J.V.d.S.); mansur@bioquimed.ufrj.br (J.M.M.); dmj.agost@gmail.com (D.M.J.A.); 2 Department of Cell Biology, University of Virginia, Pinn Hall, Charlottesville, VA 22908, USA; karinaldtexeira@gmail.com; 3 Division of Hematology, Brigham and Women’s Hospital, Harvard Medical School, Boston, MA 02115, USA; michael.chorev@harvard.edu; 4 Department of Biomolecular Sciences, University of Urbino Carlo Bo, 61029 Urbino, Italy; aurora.diotallevi@uniurb.it (A.D.);

**Keywords:** Leishmaniasis, eIF2α, translation initiation, eIF2α kinase activators, di-substituted urea-derivatives

## Abstract

Protein synthesis has been a very rich target for developing drugs to control prokaryotic and eukaryotic pathogens. Despite the development of new drug formulations, treating human cutaneous and visceral *Leishmaniasis* still needs significant improvements due to the considerable side effects and low adherence associated with the current treatment regimen. In this work, we show that the di-substituted urea-derived compounds **I-17** and **3m** are effective in inhibiting the promastigote growth of different *Leishmania* species and reducing the macrophage intracellular load of amastigotes of the *Leishmania (L.) amazonensis* and *L. major* species, in addition to exhibiting low macrophage cytotoxicity. We also show a potential immunomodulatory effect of **I-17** and **3m** in infected macrophages, which exhibited increased expression of inducible Nitric Oxide Synthase (NOS2) and production of Nitric Oxide (NO). Our data indicate that **I-17**, **3m**, and their analogs may be helpful in developing new drugs for treating leishmaniasis.

## 1. Introduction

Leishmaniasis are neglected infectious diseases caused by protozoa of the genus Leishmania, whose clinical manifestations may depend on the parasite species and the host’s immune profile, among other factors [1]. In Brazil, Cutaneous Leishmaniasis (CL) is caused by species belonging to the Sub-Genera *Viannia* and *Leishmania*, while the species *L. infantum* is the principal agent of Visceral Leishmaniasis in Europe and Brazil [2,3]. *Leishmania* infections are of great clinical relevance to humans and domestic animals. Cutaneous and visceral leishmaniasis are prominent neglected diseases worldwide, affecting hundreds of thousands of people. Liposomal amphotericin B is used in visceral leishmaniasis and mucosal leishmaniasis. However, the administration of amphotericin B requires the availability of medical facilities with professional staff. Antimonial injections may lead to undesirable side effects, and long-term administration frequently results in low adherence to the treatment. Pentamidine isethionate is a second-line treatment. Due to nephrotoxicity, this agent is mainly used in *L. guyanensis* infections [4,5]. The oral treatment of Cutaneous Leishmaniasis relies on the use of the FDA-approved drug Miltefosine [6,7]. However, the clinical efficacy varies immensely among the clinical trial results reported thus far [8]. Moreover, Miltefosine is teratogenic, which limits its comprehensive utilization [9]. The large number of *Leishmania* species involved in human infections and the impact of viral coinfections challenge the perception of the actual effectiveness of the ongoing therapeutics in the treatment of leishmaniasis.

Di-substituted urea derivatives have shown encouraging results in controlling tumor cell proliferation and xenograft tumor growth [10]. These compounds inhibit mRNA translation by inducing the phosphorylation of the translation initiation factor 2 (eIF2) subunit alpha (eIF2α) in eukaryotes [11]. eIF2α is essential for forming the ternary translation initiation complex between eIF2.GTP. Met-tRNAi is required to initiate protein synthesis in eukaryotic cells. The phosphorylation of eIF2α leads to a global attenuation of protein synthesis. Four kinases mediate this phosphorylation [12]. HRI (heme-regulated inhibitor kinase) is activated by heme deprivation in the erythroid lineage or oxidative and mitochondrial stress in other cells [13]. PERK (protein kinase R-like endoplasmic reticulum kinase) is activated by endoplasmic reticulum stress and by intracellular pathogens such as viruses and *Leishmania*, [14], GCN2 (general control nonderepressible 2) is activated by amino acid deprivation [15] and, finally, PKR (protein kinase R) is activated by double-stranded RNA, particularly in response to viral infection [16]. A high throughput screen for small molecular weight chemicals that induce eIF2α phosphorylation identified di-substituted ureas as a privileged scaffold [17]. Subsequent testing of an extensive focused di-substituted urea library, and follow-up structure–activity relationship studies, identified *N′N*-diarylurea and *N*-aryl-*N*-cyclohexyl ureas *(cHAUs)* as a specific and potent HRI activator. These agents inhibit the proliferation of all cancer cells tested, including estrogen receptor-positive MCF7 breast cancer and mutant BRAF-driven melanoma cancer cell lines [17], and inhibit xenograft tumor growth [17,18].

The phosphorylation of eIF2α is essential for the differentiation of *Leishmania*, mediated by the activation of PERK [19]. We reasoned that *N′N*-diarylureas and *cHAUs* would reduce the viability of *Leishmania* parasites by activating parasite homolog(s) of HRI, thereby attenuating parasite protein synthesis. Reduced protein synthesis should alter parasites’ proliferation and/or differentiation. When tested on trypanosomatids of the genus *Trypanosoma*, *N′N*-diarylureas and *cHAUs* inhibited parasite proliferation by decreasing the rate of infection and the number of parasites per infected cell. Among the library of compounds tested, **I-17**, an *cHAU,* was the most promising agent in reducing *T. brucei* and *T. cruzi* growth with a high specificity index [20]. **I-17** also displayed significant activity against *Listeria monocytogenes* by inhibiting pathogen trafficking [21]. We, therefore, tested a library of *N′N*-diarylureas and *cHAUs* for their potency in inhibiting *Leishmania* proliferation and infectivity. We report here that *N′N*-diarylureas and *cHAUs* potently inhibited the proliferation *of L. amazonensis*, while **NCPdCPU** [17], an *N′N*-diarylurea compound that cannot induce eIF2α phosphorylation, was without effect.

## 2. Materials and Methods

**Figure 1:**
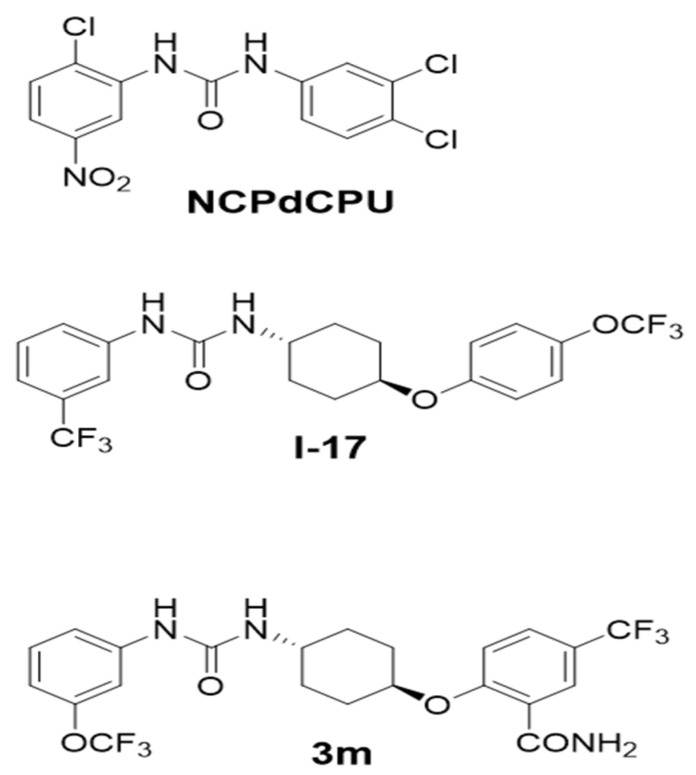
Structures of the active compounds **I-17**, **3m** and the inactive compound **NCPdCPU**.

Identification, synthesis, structure–activity relationship studies, and biological evaluation of the compounds used for these studies are described in [17,18,22,23,24]. The compounds **I-17** and **3m**, whose synthesis was originally described in [18] and [22], were purified to >98% by flash chromatography and dried as a white powder. All compounds were solubilized in DMSO as 20 mM stock solutions and aliquoted and stored at −20 °C until use. An inactive *N′N*-diarylureas analog, **NCPdCPU**, was similarly purified to >98% purity and isolated as a white powder [17]. The structures of the most active compounds **I-17** and **3m** and an inactive analog **NCPdCPU** are shown in Figure 1.

### 2.1. Cell lines and Culture Conditions

The RAW 264.7 macrophages (ATCC: TIB-71) were maintained in DMEM (GIBCO) supplemented with 10% (*v*/*v*) heath inactivated fetal bovine serum (GIBCO) and 100 U/mL penicillin and 100 mg/mL streptomycin (Invitrogen). The cells were cultured in 100 mm tissue culture plates and incubated in 10% CO_2_ atmosphere at 37 °C in a humidified incubator. Cells were passaged by trypsinization with 0.2% trypsin plus 0.5 mM EDTA.

### 2.2. Leishmania spp.

*Leishmania amazonensis*, strain WHOM/BR/75/Josefa and *L. major* (MRHO/Sv/59/P) were maintained in Schneider Insect Medium (Sigma-Aldrich, St. Louis, MO, USA) supplemented with 10% FBS (Gibco). The promastigotes formed in the stationary phase (day 5 of the culture) were used for the infections. *L. infantum* strain MHOM/TN/80/IPT1 and MHOM/IT/08/31U, as well as two clinical isolates, were also used in this study. *L. infantum* promastigotes were cultivated at 26 °C in Evans’ Modified Tobie’s Medium (EMTM). The parasites were inoculated into the footpad of BALB/C mice to maintain their virulence, and *L. infantum* strains were inoculated intraperitoneally in hamsters.

### 2.3. Viability Assays

Viability tests were performed as follows: 2 × 105 RAW 264.7 macrophages or 5 × 10^5^ *L. amazonensis*, *L. major*, and *L. infantum* promastigotes were plated and exposed to different concentrations of compounds one day later for 48 h, and the MTT (Cell Titer Proliferation Assay) or MTS (Cell Titer 96H Aqueous Non- Radioactive Cell Proliferation Assay, Promega) were used to test cell viability. The 50% cytotoxicity concentrations (CC50) (for macrophages), the 50% effective concentration (EC50), and the 90% effective concentration (EC90) (for parasites) were calculated as % cell proliferation with the following formula: % = (AT − AB)/(AC − AB) × 100, where AC is the absorbance of the untreated sample, AT is the absorbance of the treated samples, and AB is the absorbance of the blank (without cells).

### 2.4. Infection Index

To determine the Infection Index, RAW 264.7 cells were plated at a density of 5 × 10^4^ in a 24-well plate and infected with *L. amazonensis* or *L. major* (10:1) a day later. Then, 24 h after infection, cells were exposed to various concentrations of test compounds for 48 h. The infection index was calculated as follows: the percentage of infected macrophages multiplied by the number of amastigotes per macrophage [25,26]. The selectivity index (SI) was calculated from the ratio of the CC50 values in host cells to EC50 values in parasites.

### 2.5. GRIESS Test

To measure the production of Nitric Oxide (NO), RAW 264.7 cells were plated at a density of 2 × 10^5^, infected for 24 h with *L. amazonensis* (10:1) a day later, and treated with the compounds at a concentration of 5 µM. The GRIESS test was carried out as described by the manufacturer (G4410 Sigma-Aldrich)

### 2.6. RT-PCR Assays

Real-time PCR reactions were performed using the Step One Real-Time PCR System (Applied Biosystems, Waltham, WA, USA). The reactions were carried out in triplicate, using the GoTaq qPCR Master Mix kit (Promega, Madison, WI, USA) in 7.5 μL of SYBR green PCR master mix, and 1 μL of cDNA and nuclease-free water (Promega, Madison, WI, USA), in a final volume of 15 μL. The analysis was carried out using Step One version 2.0 software (Applied Biosystems, Waltham, WA, USA) using the ∆∆CT method. Primers utilized were GAPDH Forward 5′- TGCACCACCACCTGCTTAGC-3′,GAPDHReverse 5′GGCATGGACTGTGGTCATGAG- Gene ID: 14433 3′Mu-NOS2-Foward: 5′-CAGCTGGGCTGTACAAACCTT- 3 and Mu-NOS2-Reverse:5′-CATTGGAAGTGAAGCGTTTCG-3′,′ Gene ID:18126 SOD1 Foward: 5′GTCTCGAGCTCGCGACCCGAGGCTG-3′, SOD1-R: 5′-GTAGATCTCAGGAGACTACGACGCAAACCAGC-3′;Gene ID: 20655; Nrf2-Foward: 5′ AAGTCCGGGTCCCAGCTCAGAG 3′ and Nrf2-Reverse: 5′-TGGGGGCGGAACAAGGACCTAG-3′ Gene ID: 18024

### 2.7. Puromycin Incorporation Assay

*L. amazonensis* promastigotes were treated with DMSO, 5 μM of **I-17**, 5 μM of **3m**, or 10 μM of cycloheximide for 2 h, followed by an addition of 10 μM of puromycin (P4512- Sigma-Aldrich) for an additional 2 h. An equal amount of cell lysates was separated by SDS-PAGE, transferred to a nitrocellulose filter, and probed with anti-puromycin (MERCK MABE343) or anti-actin (Sigma-Aldrich A2543) antibodies. After extensive washing, blots were incubated with horse radish peroxidase-conjugated secondary antibodies, and the antibody–antigen complexes were visualized using ECL reagents.

### 2.8. Statistical Analysis

Data were analyzed by two-way analysis of variance (ANOVA) for independent samples followed by Bonferroni’s Multiple Comparison Test (with no designated control group), using GraphPad Prism 6 software (San Diego, CA, USA). Data are presented as the mean values ± standard error of three independent experiments’ mean (SEM). Comparisons between means were statistically significant with *p* < 0.05.

## 3. Results

We initially tested a library of 25 *N*,*N*′-diaryl urea and *cHAU* compounds for their effects on the survival of promastigote cultures of *L. amazonensis* at 10 μM by MTT assay, Supplementary Table S1. We then tested those compounds that inhibited parasite survival by at least 50% using a wide range of compound concentrations to determine their EC50 and EC90 values against the parasites. In parallel, we tested the same compounds for their half cytotoxic concentration, CC50 values comparing them to Pentamidine and Miltefosine in RAW 264.7 macrophages to determine cells’ sensitivity, seeking a broader window to use the compounds safely to resolve the infection without leading to macrophage death. These data are shown in Supplementary Figure S1.

The compounds’ EC50 values (Table 1) against *L. amazonensis* and *L. major* were consistent with their EC50 values against the other trypanosomatids, such as *T. cruzi* and *T. brucei* [20]. From these studies, we concluded that **I-17** and **3m** were the most promising agents in this library for further characterization. The selectivity index (SI) was calculated from the ratio of the CC50 values of host cells and the EC50 values in parasites. To further validate the activity of the selected compounds, we also tested **I-17** in four strains/clinical isolates of *L. infantum*. The results were similar to those obtained with the *L. amazonensis* and *L. major* strains (Supplementary Table S2).

**Figure 2:**
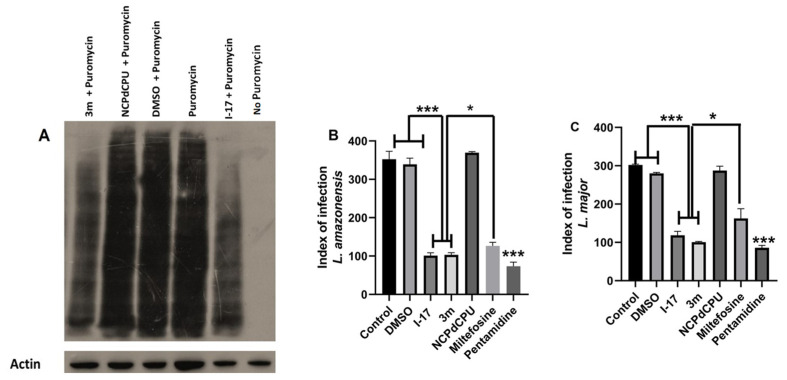
eIF2α kinase activators inhibit translation initiation and the amastigote load of *Leishmania*. (**A**) *L. amazonensis* promastigotes were treated with DMSO, 5 μM each of **I-17**, **3m** or inactive analog **NCPdCPU** for 2 h and then treated for an additional 2 hours with 10 μM of puromycin. Cells were lysed, and equal amounts of lysates were separated by SDS-PAGE and blotted using anti-puromycin or anti-actin antibodies (loading control). (**B**) Infection index. RAW cells infected with *L. amazonesis* and treated with 5 μM of **I-17**, **3m**, **NCPdCPU**, Miltefosine (40 μM) or Pentamidine (0.3 μM). DMSO-treated or not treated cells are used as controls. (**C**) The experiment in B was repeated using *L. major*. N = three independent experiments. * *p* < 0.05; *** *p* < 0.001.

We utilized a puromycin incorporation assay to determine whether **I-17** inhibited parasite proliferation by attenuating parasite protein synthesis. The inhibition of puromycin incorporation into the newly synthesized proteins, in the presence of test compounds but not in the presence of cycloheximide, is utilized as a test of whether a given compound is an inhibitor of translation initiation. As shown in Figure 2A, active compounds **I-17** and **3m**, but not inactive analog **NCPdCPU**, inhibited puromycin incorporation in newly synthesized parasite proteins. Supplementary Figure S2 shows that the inhibition of puromycin incorporation in parasite proteins is independent of the presence of cycloheximide, thus demonstrating unequivocally that the **I-17** attenuates the translation at the level of initiation in these parasites.

These results prompted us to determine the effect of **I-17** in reducing infection load in macrophages. Our studies indicate that **I-17** was similarly effective against promastigotes or intracellular amastigotes. Figure 2B,C show the effect of **I-17**, **3m**, their inactive analog **NCPdCPU**, Miltefosine, and Pentamidine on the infection index in *L. amazonensis* and *L. major*, respectively.

**Figure 3:**
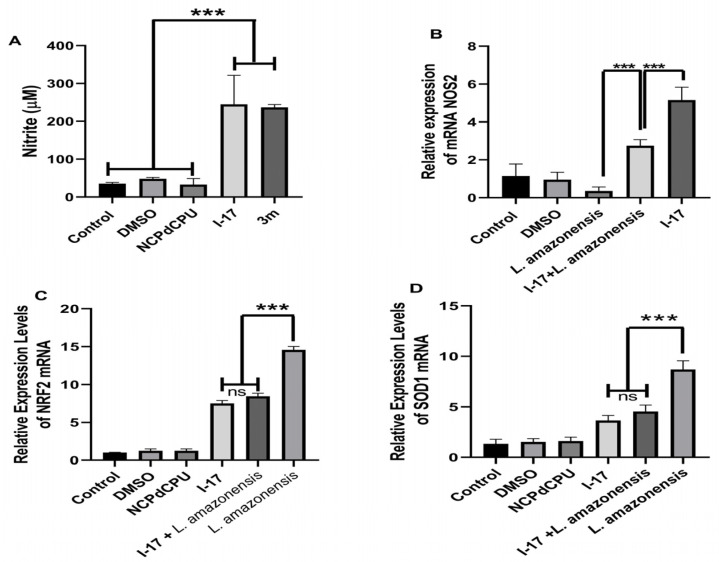
**I-17** induces the expression of NOS2 and the production of NO while inhibiting the adaptive host cell response to oxidative stress. (**A**) RAW264.7 macrophages were treated with DMSO, **I-17**, **3m**, or **NCPdCPU** and nitrile production was measured. (**B**) RAW264.7 macrophages were infected with *L. amazonensis* promastigotes and incubated with **I-17** or DMSO 24 h after infection. Uninfected cells were similarly treated and used for comparison. qPCR assays were performed with total RNA collected 24 h after treatment (5 μM), using NOS2 specific primers. (**C**,**D**) Macrophages infected and treated as in A were evaluated for the expression of Nrf-2 (**C**) or SOD1 (**D**). N = 3 independent experiments; *** *p* < 0.001. ns = not significant.

We then investigated the possible immunomodulatory effect of **I-17** and **3m** on infected macrophages. The production of Nitric Oxide (NO) is a critical factor for controlling *Leishmania* infection and depends on the induction of Nitric Oxide Synthase (NOS2) expression. We selected the dose of 5 μM to investigate whether **I-17** and/or **3m** promote NO production by inducing the expression of NOS2. As shown in Figure 3A, treatment of cells with **I-17** or **3m**, but not inactive analog **NCPdCPU**, led to NO production. Consistently, **I-17** induced NOS2 mRNA with or without infection with *L. amazonensis* (Figure 3B). Based on these data, we decided to test whether the **I-17** compound would inhibit the antioxidative-stress mediated gene response triggered by *L. amazonensis*. To address this hypothesis, we measured the relative RNA expression of the transcription factor Nrf2 (nuclear erythroid 2-related factor 2) and the enzyme SOD1 (superoxide dismutase 1). Figure 3C,D show the reduction of Nrf2 and SOD1 expression when infected cells were treated with **I-17**, but not with inactive compound **NCPdCPU**.

## 4. Discussion

Current treatment regimens for leishmaniasis must be significantly improved to reduce the treatment-associated toxicities and render them suitable for administration in areas with limited access to healthcare facilities to improve compliance [27]. The need to find new therapeutic agents and clinical approaches to treat leishmaniasis has prompted an intense search for new drug candidates to improve the treatment of the disease [10].

As proof of the principle that the eIF2α pathway can be targeted for the treatment of leishmaniasis, we tested a library of *N′N*-diarylurea and *cHAU* compounds against *Leishmaia parasites* and, using a discovery funnel, chose **I-17** for in-depth studies. Because **I-17** inhibits translation initiation rather than elongation, this allowed us to test the hypothesis that inhibitors of translation initiation will reduce promastigote replication and the infection index of *Leishmania*. Previously, we screened 25 analogs of N,N’-diarylureas and *cHAUs* against *Trypanosoma cruzi* [20]. Among these, compound **I-17** inhibited epimastigotes and intracellular amastigotes forms with a high specificity index. This class of compounds inhibits mRNA translation by activating eIF2α-kinases at the initiation stage. The screening of *N′N*-diarylurea and *cHAUs* in *L. amazonensis* and the confirmation of their activity in *L. major* and *L. infantum* demonstrated that **I-17** was the most effective compound in reducing promastigote and amastigote growth. The EC50 in promastigotes of all three *Leishmania* species ranged from 3.0 to 5.0 μM. Importantly, compounds **I-17** and **3m** effectively reduced the amastigote load in infected macrophages, slightly superior to Miltefosine.

The production of NO by infected macrophages is associated with controlling *Leishmania* infection and is one of the markers of the M1 macrophages. Cells infected with *L. amazonensis* classically show a reduction in NO, since the subversion of this pathway is important for the successful establishment of infection by the parasite [28]. Our data showed that NOS2 expression and NO production are augmented in **I-17**-treated macrophages regardless of whether they are infected or non-infected, suggesting a metabolic modulatory effect of **I-17**, which is explained by the induction of eIF2α phosphorylation favoring the generation of nitric oxide and oxidative stress in cells [18,29], a fact that corroborates previous work developed with these compounds [18]. The transcription factor Nrf2 is a key regulator of the antioxidative gene response (ARE) [30]. Previously, we have shown that, in *Leishmania* infection, Nrf2 and ARE genes are upregulated and favor the establishment of infection [31,32]. Our results show that **I-17** partially prevented the upregulation of Nrf2 and SOD1, which may contribute to infection reduction.

Our previous work showed that **I-17** treatment leads to the activation of an HRI-like kinase in *T. cruzi* and the phosphorylation of eIF2α [20]. Our mechanistic data, obtained by measuring the amino acid analog puromycin incorporation into newly synthesized proteins, revealed that **I-17** blocked *L. amazonesis* mRNA translation, most likely due to the inhibition of translation initiation. Work is underway to describe the eIF2α kinase activated in *Leishmania* by **I-17**.

## 5. Conclusions

In conclusion, the data obtained with *N′N*-diarylurea and *cHAUs*, particularly **I-17**, support the notion that these agents activate an eIF2α kinase that leads to the inhibition of translation initiation in parasites and induces NO production in host cells, developing a hostile milieu for the growth of intracellular amastigotes. The high SI justifies further in vivo studies to test **I-17** and may pave the way for developing more effective analogs against *Leishmania* based on *N’N*-diarylurea and *cHAUs* chemotypes.

## Figures and Tables

**Table 1:** Determination of EC50, EC90, and SI values of the compounds used in this study.

	*L. amazonensis*			*L. major*			
Compound	EC_50_μM	EC_90_μM	SI	EC_50_μM	EC_90_μM	SI	Reference
**I-17**	3.18	28.62	7.9	3.35	30.15	7.5	[22]
**3m**	3.26	29.34	7.4	3.48	31.32	6.9	[18]
**I-18**	4.12	37.08		4.82	43.38		[22]
**3n**	3.91	35.19		3.914.81	40.59		[18]
**3c**	3.89	35.01		4.59	44.1		[18]
**3j**	4.15	37.35		4.35	39.15		[18]
**3k**	4.02	36.18		4.41	39.30		[18]
**3l**	4.14	37.26		4.39	39.48		[18]
**3s**	4.11	36.99		4.71	39.41		[18]
**3t**	3.86	34.74		3.97	35.43		[18]
**NCPdCPU**	80.51	264.078		83.47	751.23		[17]
**Pentamidine**	0.16	1.44	147.5	0.29	2.61	81.4	
**Miltefosine**	28.38	254.7	1.8	32.45	292.35	1.6	

## Data Availability

All the research data are available from the corresponding authors upon request.

## Supplementary Materials

The following supporting information can be downloaded at: https://www.mdpi.com/article/10.3390/pathogens13020104/s1, Supplementary Table S1: Inicial screening of *L. amazonensis* promastigote proliferation by di-substituted urea compound at 10 μM; Supplementary Table S2: Effective concentration of compounds in different strains of *L. infantum*; Supplementary Figure S1: RAW Cytotoxicity Assays: Comparison of the cytotoxic concentration (CC50) of di-substituted urea compounds compared to miltefosine and pentamidine, which are compounds used in the treatment of leishmaniasis; Supplementary Figure S2: eIF2a kinase activators inhibit *Leishmania* translation at the initiation stage. *L. amazonensis* promastigotes were treated with, DMSO, 5 μM of **I-17**, **3m** for 2 h and then treated for additional two hours with 10 μM of puromycin in the presence or absence of cycloheximide. Cell were lysed and equal amounts of lysates were separated by SDS-PAGE and blotted using anti-puromycin or anti-actin antibodies (loading control).
