# Supplementary material for: Activity of the Di-Substituted Urea-Derived Compound I-17 in Leishmania In Vitro Infections
Source: Pathogens. 2024 Jan 24;13(2):104. doi: 10.3390/pathogens13020104 (PMC10893125; doi:10.3390/pathogens13020104)
Supplement: Supplementary file 1 [file pathogens-13-00104-s001.zip › pathogens-2776289-supplementary.pdf]

Compound	% inhibition	reference
3p	48	[18]
6a	41	[18]
**I-17**	**87**	**[22]**
3e	37	[18]
6b	35	[18]
**I-18**	**70**	**[22]**
3g	42	[18]
3r	38	[18]
3q	39	[18]
3f	43	[18]
3o	36	[18]
**3m**	**83**	**[18]**
3i	45	[18]
I-m6	30	[22]
3a	44	[18]
**3c**	**68**	**[18]**
3h	41	[18]
3b	26	[18]
**3n**	**73**	**[18]**
3d	24	[18]
**3j**	**71**	**[18]**
**3k**	**67**	**[18]**
**3l**	**74**	**[18]**
**3s**	**65**	**[18]**
**3t**	**69**	**[18]**
**NCPdCPU**	**3**	**[17]**

Supplementary Table S1: Inicial screening of *L. amazonensis* promastigote proliferation by di-substituted urea compound at 10 µM. Results were obtained through viability tests of *L. amazonensis* promastigotes treated with 25 compounds.

	E_{C50} µM	EC_{90} µM	SI
***L. intantum* (Promastigote MHOM/TN/80/IPT1)**	3.5	31.50	13.03
***L. intantum* (Promastigote MHOM/IT/08/31U)**	4.9	9.18	9.31
***L. intantum* (Promastigote isolate 1)**	4.4	44.10	10.36
***L. intantum* (Promastigote isolate 2)**	3.3	29.70	13.82

Supplementary Table S2: Effective concentration of compounds in different strains of *L. infantum*. Results obtained through viability tests of *L. infantum* promastigotes treated with I-17

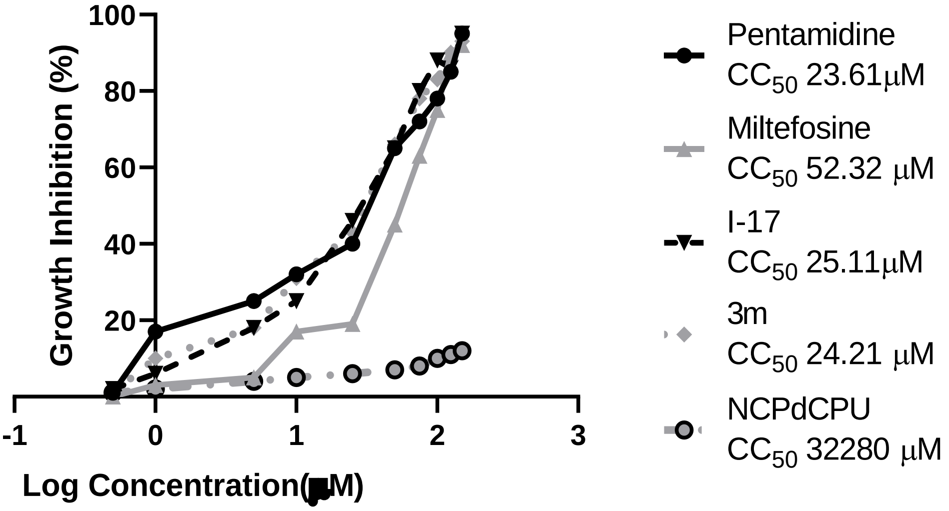

Supplementary Figure S1: RAW Cytotoxicity Assays: Analysis of di-substituted urea compounds' cytotoxicity concentration (CC50) compared to miltefosine and pentamidine.

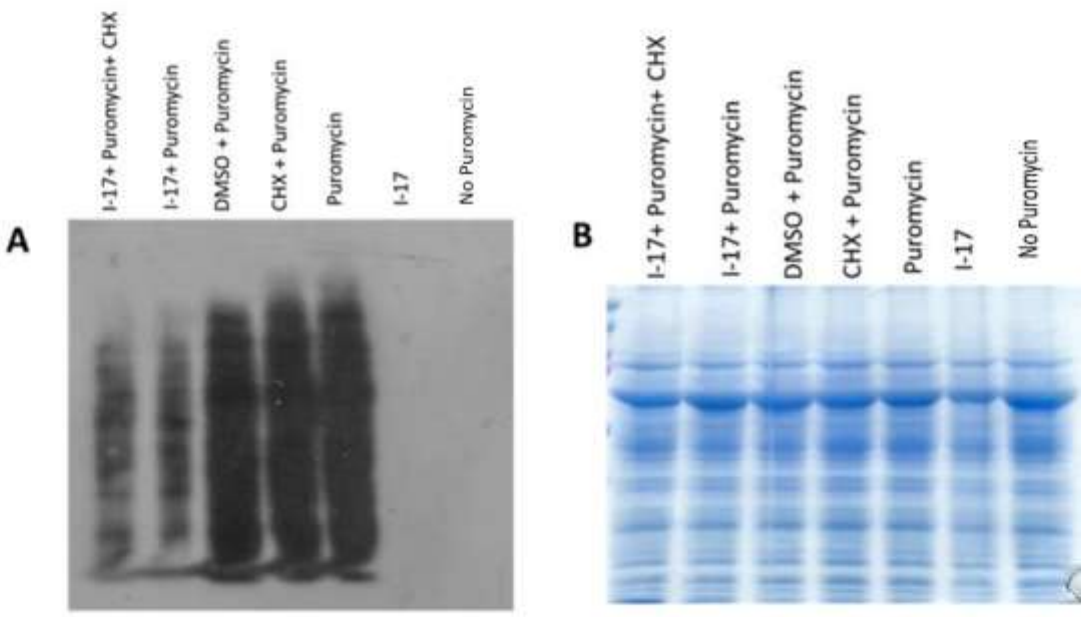

Supplemental Figure S2. eIF2a kinase activators inhibit Leishmania translation at the initiation stage. A) *L. amazonensis* promastigotes were treated with DMSO 5µM of I-17 for 2 hours and then treated for an additional two hours with 10 µM of puromycin in the presence or absence of cycloheximide. Cells were lysed and equal amounts of lysates were separated by SDS-PAGE and blotted using anti-puromycin antibodies. **B)** The same quantities of proteins were separated by SDS-PAGE and stained with Coomassie brilliant blue to ensure equal loading.
